# Prognostic Value of Spot Urinary Creatinine Concentration and Its Relationship with Body Composition Parameters in HF Patients

**DOI:** 10.3390/biomedicines11051429

**Published:** 2023-05-12

**Authors:** Jolanta Malinowska-Borowska, Małgorzata Piecuch, Patryk Szlacheta, Aleksandra Kulik, Jacek Niedziela, Jolanta Urszula Nowak, Łukasz Pyka, Mariusz Gąsior, Piotr Rozentryt

**Affiliations:** 1 Department of Chronic Diseases and Civilization-Related Hazards, Faculty of Public Health in Bytom, Medical University of Silesia in Katowice, 41-902 Bytom, Poland; 2 Department of Cardiology, Faculty of Medical Sciences in Zabrze, Silesian Centre for Heart Disease, Medical University of Silesia, 41-800 Zabrze, Poland

**Keywords:** spot urine creatinine, body composition, fat-free mass, heart failure

## Abstract

Background: Low 24-h urinary excretion of creatinine in patients with heart failure (HF) is believed to reflect muscle wasting and is associated with a poor prognosis. Recently, spot urinary creatinine concentration (SUCR) has been suggested as a useful prognostic factor in selected HF cohorts. This more practical and cheaper approach has never been tested in an unselected HF population. Moreover, neither the relation between SUCR and body composition markers nor the association of SUCR with the markers of volume overload, which are known to worsen clinical outcome, has been studied so far. The aim of the study was to check the prognostic value of SUCR in HF patients after adjusting for body composition and indirect markers of volume overload. Methods: In 911 HF patients, morning SUCR was determined and body composition scanning using dual X-ray absorptiometry (DEXA) was performed. Univariable and multivariable predictors of log SUCR were analyzed. All participants were divided into quartiles of SUCR. Results: In univariable analysis, SUCR weakly correlated with fat-free mass (R = 0.09, *p* = 0.01). Stronger correlations were shown between SUCR and loop diuretic dose (R = 0.16, *p* < 0.0001), NTproBNP (R = −0.15, *p* < 0.0001) and serum sodium (R = 0.16, *p* < 0.0001). During 3 years of follow-up, 353 (38.7%) patients died. Patients with lower SUCR were more frequently female, and their functional status was worse. The lowest mortality was observed in the top quartile of SUCR. In the unadjusted Cox regression analysis, the relative risk of death in all three lower quartiles of SUCR was higher by roughly 80% compared to the top SUCR quartile. Apart from lower SUCR, the significant predictors of death were age and malnutrition but not body composition. After adjustment for loop diuretic dose and percent of recommended dose of mineralocorticoid receptor antagonists, the difference in mortality vanished completely. Conclusions: Lower SUCR levels in HF patients are associated with a worse outcome, but this effect is not correlated with fat-free mass. Fluid overload-driven effects may link lower SUCR with higher mortality in HF.

## 1. Introduction

The global epidemic of heart failure (HF) has become an increasing medical, social and economic problem of the developed world [1]. Despite advances in therapy, mortality remains unacceptably high, the cost of treatment increases, and patients living with HF still suffer from a profound impairment of quality of life and loss of independence in daily life [2,3].

The main reason for the limited quality of life in HF patients is exercise intolerance—one of the key hallmarks of HF. Dyspnoea and fatigue occurring during effort and, in advanced stages, present even at rest, have a complexed pathophysiology involving both central and peripheral mechanisms [4]. Since the late 1980s, there has been increasing awareness of the role of skeletal muscle pathology in the exercise intolerance of HF. Numerous morphologic, structural, biochemical, and metabolic alterations have already been described [5,6,7,8]. All these abnormalities lead to the reduction of exercise capacity [9,10] and poor prognosis [11]. Thus, the analysis of the quantity and quality of muscles has become an important clinical issue in HF.

Different methods have been proposed for the assessment of skeletal muscle mass and function. The muscle mass and, to some extent, also the structure can be quantified by magnetic resonance, computerized tomography, or dual-energy X-ray absorptiometry (DEXA). However, these techniques are expensive and impractical in clinics, and they do not test the muscle function [12].

Apart from tests measuring muscle strength, there is an increasing interest in easy-to-perform biochemical analyses reflecting both functional and morphological aspects of skeletal muscles. The attempts to use creatine as a marker of muscle mass have a convincing biochemical background and a long history [13]. The availability of ATP to maintain myofilament cross-bridge cycling by myosin ATPase in skeletal muscle is necessary for muscle contraction [14]. Because the intramuscular stores of ATP is small, the phosphocreatine system is needed for fast and effective replenishment of the molecule. After restoring ATP from ADP by phosphocreatine, free creatine, a product of muscular creatine kinase, occurs in the circulation [15]. After conversion to creatinine, the molecule is excreted in urine. Almost 95% of all body creatine is synthesized in the skeletal muscles [16]. Thus, it is believed that in a steady state, the rate of creatinine excretion into urine may serve as a proxy not only of skeletal muscle mass, but also functional integrity [17].

Among biochemical tests based on the urinary excretion of creatinine, the spot urinary creatinine concentration (SUCR) analysis has recently attracted significant attention due to its simplicity and association with prognosis [18,19]. However, neither of studies have analyzed the relationship between SUCR and direct measures of body composition. Morning SUCR is also dependent on the ability of kidneys to adjust diuresis volume and its quality in states of fluid overload, which is impaired in HF and is clinically reflected by the need for higher dosages of loop diuretics and/or multidrug therapy to maintain euvolemia [20]. Therefore, in our study, we tried to establish whether presence of malnutrition in HF, the variation in body composition assessed by DEXA, and the use of higher doses of loop diuretics and aldosterone antagonists independently modify the risk of SUCR-related mortality risk.

## 2. Methods

### 2.1. Study Group

Data collected in the Prospective Registry of Heart Failure implemented in our department were used to formulate the final study cohort. All participants were recruited in an outpatient hospital setting from January 2004 to March 2013. All patients in the Prospective Heart Failure Registry were referred to our department as candidates for heart transplantation, so they all underwent several specific procedures. One of them was DEXA scanning, needed as a screening of osteoporosis and sarcopenia. Spot urinary creatinine concentration was also a part of this examination because albuminuria expressed as an albumin-to-creatinine ratio was routinely used in candidate recruitment for heart transplantation.

We selected patients with HF and a reduced left-ventricle ejection fraction (LVEF) ≤ 40%, diagnosed according to criteria published by the European Society of Cardiology, aged >18 years and with HF duration of more than 6 months. They all needed to be on their best-tolerated medical therapy, for whom HF could be confirmed with 1-month precision and with available records concerning body weight before the first diagnosis of HF and minimal weight during HF.

The onset of HF was defined as a month in which medical records prepared by a cardiologist in an outpatient setting demonstrated the coexistence of LVEF ≤ 40% with typical signs and/or symptoms of HF. The maximum unchanged therapy had to be longer than 1 month before the index date. The maximal body weight was defined based on the outpatient medical records as the highest weight within a year, but not later than 2 months before HF diagnosis. On the contrary, the lowest body weight was defined as the minimum body weight when the attending cardiologist did not change diuretics or did not perceive signs and/or symptoms of fluid retention upon clinical examination.

**Figure 1:**
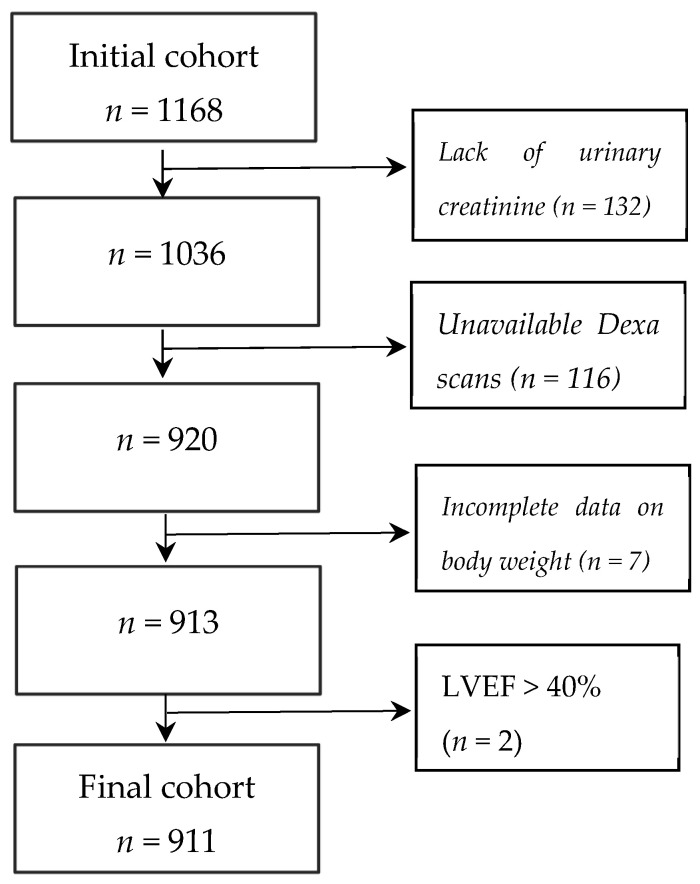
A flowchart of the final cohort.

Patients having active infection, liver disease with enzymes levels four times higher than normal, active bleeding, known neoplasm, or who had undergone bariatric surgery or surgery reducing intestinal absorptive capacity were excluded. Of 1168 registered participants, 911 fulfilled the study criteria (Figure 1). Medical records of this study group were reviewed, and comorbidities such as hypertension, diabetes mellitus, and hypercholesterolemia were recognized based on the clinical history, current medication, or actual measurements of the respective variables. A history of smoking was defined as the current or previous use of tobacco products.

One spot-urine sample was collected per person on the index day. Blood samples were drawn in a standardized manner in the morning between 8 and 10 a.m. from patients who had been fasting for at least 8 h and resting in a supine position in a quiet, environmentally controlled room for 30 min. Blood was immediately centrifuged at 4 °C and stored at −75 °C for further analysis. All procedures were undertaken in accordance with the Helsinki Declaration. The protocol was reviewed and accepted by the Ethics Committee of Medical University of Silesia in Katowice (NN-6501-12/I/04). All patients expressed their informed, written consent.

Body mass and height were measured on the index date using a certified scale (Radwag, Poland). Body mass index (BMI) was calculated by dividing weights in kilograms by heights in meters squared. PreHF BMI, min HF BMI, and index BMI corresponding to maximal, minimal, and index weights were defined in this study.

For the diagnosis of malnutrition, the GLIM criteria consist of at least one phenotypic criterion and one etiologic criterion [21]. In the present study, we diagnosed malnutrition based on phenotypic criteria. Weight loss, low preHF BMI, and reduced muscle mass were categorized as phenotypic criteria. PreHF BMI < 20 kg/m^2^ was defined as low BMI if patients were younger than 70 years, and BMI < 22 kg/m^2^ was defined as low BMI for those aged 70 years or older. Cut-off points for reduced muscle mass (ASMI < 5.5 kg/m^2^ for women and <7.26 kg/m^2^ for men) were used to determine muscle wasting. Since all patients suffered from HF, they had already met one etiologic criterion (disease burden).

The Sonos-5000 Hewlett-Packard Ultrasound Scanner; (Hewlett-Packard, Andover, MA, USA) was used to measure LVEF from the apical four-chamber view and calculate it with the following formula:

*LVEF = [(end-diastolic volume − end-systolic volume)/end-diastolic volume] × 100*

Body composition analysis was performed with the use of dual X-ray absorptiometry (DEXA) with a pencil-beam Lunar DRX-L device (General Electric, Brussels, Belgium). Compartments of body mass were measured and used in further analyses. Commercially available reagents and automatic methods (Roche Diagnostics, Switzerland) allowed to measure of hemoglobin, serum creatinine, high-sensitivity C-reactive protein, N-terminal pro-brain natriuretic peptide (NTproBNP), and serum sodium. Spot urinary creatinine concentration was measured (SUCR) for each patient. GFR was calculated from the MDRD formula:

*eGFR_MDRD_ = 186 × plasma creatinine [mg/dL]^−1.154^ × age [years]^−0.203^ × 0.742 (if female)*

### 2.2. Statistical Analysis

Categorical variables are presented as percentages. Quantitative normally distributed data are presented as means and standard deviations, whereas non-normally distributed data are presented as medians and interquartile ranges (IQR). First, an univariable analysis was performed. Then, a multivariable model was built using significantly associated variables with SUCR.

The study group was split into quartiles of SUCR. Groups were compared using Kruskal–Wallis or chi-square tests where appropriate. Kaplan–Meier survival curves for each group were also compared. Cox method was used to estimate the relative risk of death for each group according to SUCR, taking quartile 4 with lowest mortality rate as a reference. Cox models were adjusted in a stepwise manner for relevant confounders, among which were fat-free and fat mass based on DEXA, both indexed to the body surface area, markers of malnutrition, and of indirect markers of higher fluid overload. The statistical significance was set at *p* = 0.05. Statistical analyses were performed with the use of Statistica 13.3 (Statsoft, Kraków, Poland).

## 3. Results

In the group of 911 HF patients, the majority were men (85.8%). Most of the patients had HF of ischemic etiology (61.6%; *p* < 0.0001) and were in NYHA classes II and III (*p* < 0.0001). The median SUCR was 1.087 g/L (the *interquartile range (IQR):* 0.573–1.633). The detailed characteristics of the cohort included in the study are shown in Table 1.

### 3.1. Comparison of Study Groups According to SUCR

A comparison of the SUCR quartiles allowed for the identification of several differences. In quartiles with lower SUCR, there were more females. These patients also had a higher NYHA class and lower blood pressure. They lost more weight since the onset of HF, resulting in a lower index weight. Both total and appendicular fat-free tissue contents were reduced in lower SUCR subgroups. Furthermore, more patients in lower quartiles were malnourished according to the GLIM criteria (Table 1).

Comparison of laboratory results between SUCR quartiles demonstrated reduction of hemoglobin and serum sodium but higher values of NTproBNP in lower quartiles of SUCR. The distribution of comorbidities across quartiles of SUCR was similar with the exception of diabetes, which was more prevalent in patients in lower quartiles of SUCR. There were significant differences in treatment between SUCR quartiles. Patients in lower quartiles were less likely to receive the renin–angiotensin system antagonist, but more likely to be treated with mineralocorticoid antagonists and loop diuretics. The doses of loop diuretics administered to these patients were also higher.

During the 3 years of follow-up, 353 (38.7%) patients died (all-cause) in all cohorts. The risk of death was different in the SUCR groups. All three lower quartiles of SUCR showed pretty similar mortality at 3 years, while in the top quartile of SUCR, the mortality was distinctly lower.

**Figure 2:**
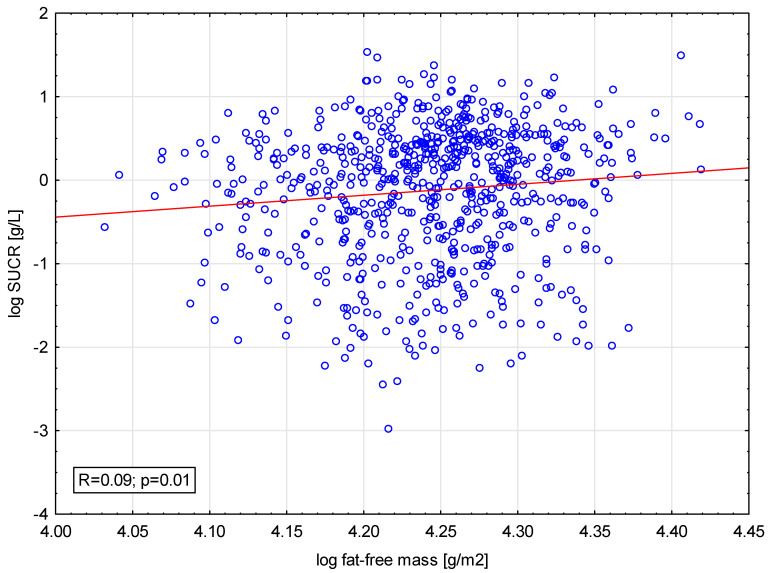
Correlation between fat-free mass and SUCR (log-transformed values).

There was only a weak but significant correlation between SUCR and fat-free mass (Figure 2). Numerous parameters correlated with SUCR on univariable analysis; however, only male gender and the presence of malnutrition by GLIM score were significant in multivariate regression analysis (Table 2). All parameters used in multivariable analysis could explain only 6.3% variability of SUCR.

**Figure 3:**
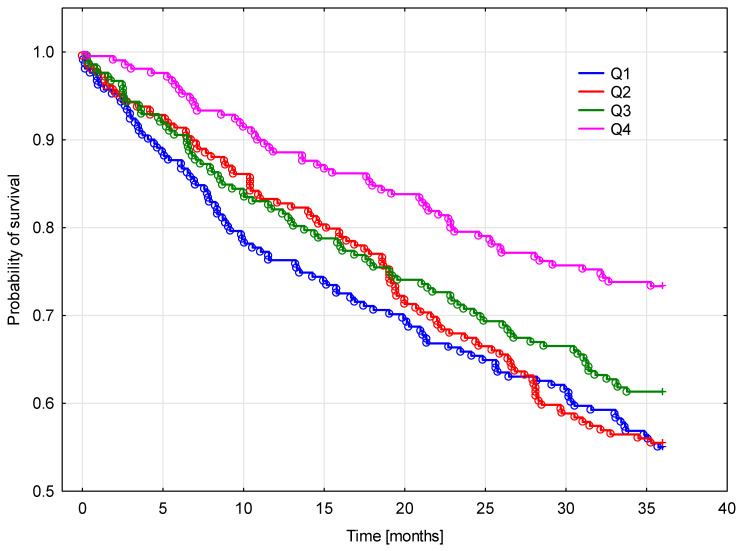
Kaplan–Maier survival probability up to 3 years according to quartiles of SUCR.

Kaplan–Meier analysis confirmed worse survival in patients with low SUCR in comparison to high SUCR (*p* = 0.001) (Figure 3).

### 3.2. The Risk of Death in Unadjusted and Adjusted Analyses

**Figure 4:**
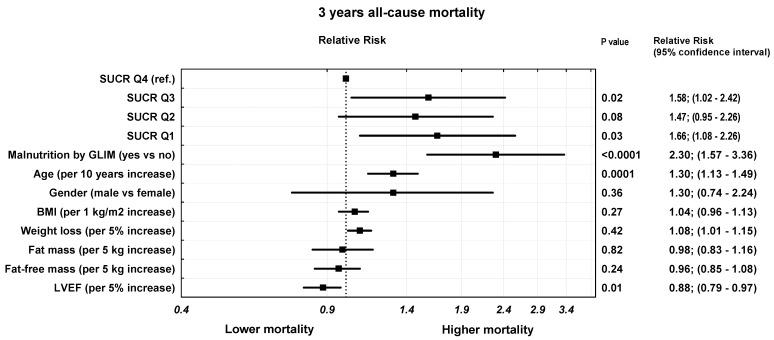
Adjusted hazard ratios (model 1) for death (all-cause) during 3 years of follow-up with quartile 4 of SUCR as a reference.

In the unadjusted model, the risk of death in all three lower quartiles of SUCR was roughly 80–90% higher than in top quartile of SUCR (Table 2). After adjustment for age, sex, BMI, weight loss, fat and fat-free tissue content, GLIM score, and LVEF, the risk in these quartiles was still increased by approximately 55% compared to the risk in the top quartile. In this model, significant predictors of death were also age: HR per 10-year increase = 1.30 (95% CI: 1.13–1.49), *p* = 0.0001; presence of malnutrition by GLIM: HR = 2.30 (95% CI: 1.57–3.36), *p* < 0.0001; weight loss: HR per 5% increase = 1.08 (95% CI: 1.01–1.15), *p* = 0.04; and LVEF: HR per 5% higher = 0.88; (95% CI: 0.79–0.97), *p* = 0.01. Body composition parameters as well as indexBMI were not predictive of the study outcome (Table 3, model 1) (Figure 4).

When we further adjusted our model by loop diuretics dose expressed as an equivalent of furosemide and by the percent of recommended dose of mineralocorticoid antagonists, the risks attributable to lower quartiles of SUCR were largely reduced and became insignificant (Table 3, model 2). The only significant predictors of the study outcome in this model were age: HR per 10-year increment = 1.37 (95% CI: 1.19–1.58, *p* < 0.0001); loop diuretics dose: HR per 40 mg of furosemide equivalent increase = 1.21 (95% CI: 1.13–1.29, *p* < 0.0001); and GLIM-based presence of malnutrition: HR = 1.87 (95% CI: 1.27–2.75, *p* < 0.0001). Neither composition variables, weight loss, or LVEF were predictive of death.

In our final model, we additionally adjusted for log NTproBNP and serum sodium. In this analysis, the risk difference between SUCR quartiles was similar to a previous model (Table 3, model 3), while significant predictors of death were still age: HR per 10-year increment = 1.32 (95% CI: 1.16–1.52, *p* < 0.0001); the dose of loop diuretics: HF per 40 mg of furosemide equivalent increase 1.15 (95% CI: 1.07–1.24, *p* = 0.001); and NTproBNP: HF per 1 log increase = 3.07 (95% CI: 2.09–4.52, *p* < 0.0001); and presence of malnutrition by GLIM: HF = 1.63; (95% CI: 1.10–2.40, *p* < 0.01).

As there was no clear risk increment in three descending quartiles of SUCR, we attempted to identify the threshold level of SUCR below which the risk is elevated. In ROC analysis, we identified the SUCR level of 1.34 g/L as optimally discriminating the dead from the alive.

## 4. Discussion

Previous research firmly established that under stable renal function, diet, and physical activity, a 24 h urinary excretion of creatinine may serve as a reliable marker of muscle mass [22] and provide important prognostic information in different clinical scenarios [23,24,25], including HF [26]. However, reliable urine collection is time consuming and may be problematic in patients with severe HF. Thus, spot urinary creatinine concentration has recently attracted attention in HF as a potential easy and inexpensive alternative. The most important observation from our study is the link between low SUCR-related mortality and more advanced congestion.

Only few studies published so far have analyzed the determinants and also the prognostic value of SUCR in HF [18,19,27]. The comparison of these studies is cumbersome as the cohorts recruited vary. The earlier research reported data on patients included in GISSI-HF and BIOSTAT-HF clinical trials in which participants were highly selected based on respective inclusion/exclusion criteria. They recruited HF patients with symptomatic congestion, while therapy used at the date of inclusion was to be optimized later on.

On the contrary, more recent work reported data from our clinical registry, enrolling an unselected “real life” cohort. Compared to previous reports, our patients were 10 years younger on average, with a higher proportion of males; free of congestive symptoms or signs; and on the maximum therapy recommended by guidelines [27].

Despite these dissimilarities and a striking variation in median SUCR, all studies consistently found the association of lower SUCR with more severe symptoms and worse prognosis [18,19,27]. Additionally, two studies reported the association between higher degree of body wasting and lower SUCR [19,27]. In one study, patients in lower SUCR subgroups also had lower appendicular fat-free mass (total fat-free mass was borderline, *p* = 0.06) [27].

Until now, no studies have directly compared the prognostic value of 24 h urinary excretion of creatinine with SUCR or analyzed body composition markers assessed with reliable techniques as potential determinants of SUCR and adjusted SUCR-related mortality for fat-free content.

Our current report is the first to show that SUCR levels only weakly correlate with fat-free tissue content measured by DEXA, and the prognostic power of low SUCR in HF is independent of this body composition compartment. Additionally, we found a negative correlation between SUCR and the loop diuretic dose necessary to maintain the euvolemic state. This correlation was stronger than between SUCR and fat-free mass. The stronger correlation was also present between SUCR and NTproBNP or serum sodium, both known to reflect sodium and water excess. It is worth noting that SUCR levels also correlated with the percent of recommended dose of the mineralocorticoid antagonist. This finding may be an additional argument suggesting the link between SUCR and tissue congestion because higher dosages of mineralocorticoid antagonists prompt higher natriuresis, thereby allowing maintenance of euvolemia in patients with stronger tendencies to generate congestion [28].

The association of mortality with lower SUCR shown after adjustment for age, gender, BMI, weight loss, body composition, and nutritional markers vanished when we included our surrogate of congestion with the percent of recommended dose of mineralocorticoid antagonist or when excess water, sodium, or both were added to the multivariable model.

These findings suggest that low SUCR cannot serve as a direct marker of muscle wasting. Conversely, lack of a significant association between lower SUCR and mortality after adjustment for loop diuretic dose, percent of recommended mineralocorticoid antagonists, and further for NTproBNP and serum sodium—markers of water and sodium overload—may argue that congestion, even as documented based on surrogate markers, may be a primary, upstream event leading to low SUCR.

Such a hypothesis would explain why in all studies, lower SUCR levels aggregated with more severe HF symptoms, more body wasting, and worse outcomes. Numerous studies published to date have convincingly linked congestion with more advanced symptoms, malnutrition, body wasting, and worse outcomes [29,30,31].

The SUCR level does not directly reflect the urinary creatinine excretion rate. It represents a dynamic balance between the production, absorption, and breakdown of creatine from which creatinine originates, as well as the ability of the kidney to excrete creatinine and to dilute or concentrate the final urine. Each of these processes can by modified by the presence of congestion and by treatment.

Approximately 50% of the daily requirement for creatine necessary to synthesize of phosphocreatine and maintain energetic metabolism is endogenously synthesized in the liver, while remaining part comes from the diet [32]. Thus, in liver and gut congestion, creatine production or dietary supply and absorption from the gut may be impaired, reducing the creatinine pool for excretion. Thus, in systemic congestion, many factors may work to lower the SUCR level.

Data from previous studies support this hypothesis. The higher congestion burden was reported in these studies; the lower were medians of SUCR. Congestive signs and symptoms were seen in 79.6% of patients in BIOSTAT-HF and in 33.1% of participants in GISSI-HF. Patients participating in our previous and current study were free of congestion [27]. The respective medians of SUCR were 0.588 g/L, 0.8 g/L, 1.04 g/L, and 1.09 g/L [18,19,27]. As creatinine is produced mainly in muscles [33], one may argue that a higher SUCR in two more recent studies may be related to a higher fat-free tissue content due to the fat-free tissue content because the study group is 10 years older. However, it is unlikely to be true because longitudinal observations show that the loss of skeletal muscle mass during the 10 years was <10% [34]. We feel that even slightly higher proportions of men—and hence, potentially higher production of creatinine in our cohorts—does not fully explain the 20–40% difference of the medians of SUCR.

In normal subjects, SUCR levels vary, mainly due to the specific gravity of urine and urine volume flow [35]. The specific urine gravity is the measure of a kidney’s ability to adjust urine volume through concentration or dilution to maintain normal fluid volume and osmolality. Neither of the studies have measured urine gravity specifically.

There are numerous clinical scenarios in which kidneys may produce lower-gravity urine, and hence, lower SUCR with elevated volumes. Patients with systemic congestion have a rostral fluid shift in the supine position, thus promoting the increased nighttime production of lower-gravity urine [36]. In fact, nocturia is common in HF, linked to volume overload, breathing abnormalities, worse functional status, and elevated natriuretic peptides [37,38]. Even in the community-dwelling population, increased levels of NTproBNP—an indicator of elevated volume stress on the heart—have recently been shown to raise the risk of nocturia as high as 3–7 times [39]. In fact, our study showed a negative correlation between NTproBNP and SUCR. The higher volumes of low-gravity urine can also be produced in patients with an elevated osmotic load due to diabetes, when the osmotic load per nephron is increased as in cases of reduced kidney function, and in patients receiving higher dosages of diuretics [40,41].

In all reports concerning SUCR levels in HF, patients with lower SUCR were more likely to have a clinical situation that promoted the production of less-concentrated urine with higher volumes. Patients with lower SUCR had more evident features of congestion (in GISSI-HF and BIOSTAT-HF), had higher natriuretic peptides, had worse kidney function, received more diuretics, and more frequently had diabetes [18,19,27]. Therefore, it is quite possible that these factors contributed significantly to a lower SUCR.

In older reports, significant negative predictors of SUCR were symptoms and signs of clinical congestion, again arguing for low SUCR as a marker of uncorrected or unrecognized congestion rather than as a marker of lower skeletal muscle mass.

## 5. Limitations

Our study has several important limitations. The retrospective nature of study precludes any inference on causality. We did not assess appetite and diet in our patients, so we cannot exclude differences of these factors as significant contributors to low SUCR. No evaluation of muscle function is also a limitation. Neither direct measurement of urine-specific gravity nor urine volumes, particularly voided during urine sampling for analysis, were available; therefore, higher volumes with lower gravity assumed as a potential reason for lower SUCR levels cannot be indisputably confirmed. Important information on loop diuretics dosage timing was also lacking.

## 6. Conclusions

Our data show that lower SUCR levels in HF patients are associated with worse outcomes. Contrary to expectations, however, lower SUCR was not correlated with fat-free mass. Fluid overload-driven effects may link lower SUCR with higher mortality in HF, and this tempting hypothesis warrants further studies. If confirmed in future research, this cheap and easy obtainable marker could be added to the arsenal of measures useful for the monitoring of congestion relief in everyday clinical practice.

From a clinical standpoint, the key innovation of the study is that in the case of a low SUCR value, clinicians should take into account a presence of undiagnosed and/or uncorrected congestion as an underlying factor. Our data suggest that low SUCR may be a marker of congestion beyond the standard parameters assessed so far. From a scientific point of view, the study justifies further research on the relative value of SUCR versus other markers of congestion.

## Figures and Tables

**Table 1:** Clinical and laboratory characteristics of all patients and subgroups after splitting into quartiles of spot urinary creatinine (means ± SD, medians, IQR).

		Quartiles of Spot Urinary Creatinine [g/L]				
Feature	All *n* = 911	Q1 *n* = 227 (0.050–0.573)	Q2 *n* = 228 (0.573–1.087)	Q3 *n* = 228 (1.087–1.633)	Q4 *n* = 228 (1.633–5.058)	*p* Value
**Baseline Demographics and Functional Tests**						
Age (years)	52.9 ± 11	52.4 ± 11	52.6 ± 11	53.4 ± 11	53.1 ± 9	0.74
Males (%)	86	81	82	87	93	<0.0001
HF etiology–ICM (%)	62	62	58	64	62	0.65
NYHA class	2.6 ± 0.8	2.8 ± 0.8	2.8 ± 0.8	2.5 ± 0.8	2.4 ± 0.7	<0.0001
NYHA class I/II/III/IV (%)	6/37/48/9	6/25/55/14	5/29/51/14	7/40/43/9	10/46/41/3	<0.0001
Duration of HF (months)	34.4 (14–70)	27 (12–66)	41 (14–77)	34 (13–68)	39 (15–39)	0.22
Systolic BP (mmHg)	109 ± 16	107 ± 16	106 ± 16	111 ± 17	110 ± 16	0.002
Heart rate (beat per minute)	82 ± 15	81 ± 14	81 ± 13	82 ± 16	83 ±15	0.57
MVO_2_ (mL/kg min)	15.0 (12.2–18.4)	14.9 (11.9–18.6)	14.7 (11.9–18.6)	14.7 (12.3–17.9)	15.3 (12.6–18.6)	0.60
LVEF (%)	25 ± 8	25 ± 8	25 ± 9	26 ± 9	26 ± 8	0.50
**Anthropometrics and Body Composition**						
PreHF BMI (kg/m^2^)	28.2 ± 4.6	28.0 ± 4.7	28.0 ± 4.6	28.3 ± 4.8	28.5 ± 4.3	0.59
IndexBMI (kg/m^2^)	26.3 ± 4.5	25.4 ± 4.2	25.7 ± 4.4	26.8 ± 1.9	27.4 ± 4.2	<0.0001
Weight loss from preHF BMI until index BMI (%)	11.0; (4.9–17.6)	13.8; (6.7–19.4)	11.2; (5.5–18.6)	9.2; (3.5–16.5)	9.0; (3.1–14.6)	<0.0001
Fat mass (kg/m^2^)	7.2; (5.6–9.0)	6.9; (5.0–8.7)	7.2; (5.7–8.8)	7.3; (6.0–9.1)	7.6; (5.8–9.3)	0.06
Fat-free mass (kg/m^2^)	17.7; (16.0–19.4)	17.4; (15.7–19.1)	17.5; (15.5–19.4)	17.6; (16.2–19.2)	18.3; (16.8–19.9)	0.0002
**Laboratory Tests**						
Hemoglobin (mmol/L)	8.7 ± 1.1	8.6 ± 1.1	8.8 ± 1.0	8.6 ± 1.1	8.8 ± 1.0	0.04
NTproBNP (pg/mL)	1375; (647–3069)	1598; (805–3690)	1644; (693–3477)	1356; (638–2982)	1081; (447–2293)	0.008
eGFR_MDRD_ (mL/min × 1.73 m^2^)	86; (55–105)	83; (63–106)	88; (65–1053)	88; (70–107)	83; (66–101)	0.52
eGFR_MDRD_ < 60 mL/min × 1.73 m^2^ (%)	22.2	21.1	21.9	14.9	15.3	<0.05
Sodium (mmol/L)	134; (136–138)	136; (133–138)	136; (134–138)	136; (134–138)	135; (138–139)	<0.001
hCRP (mg/dL)	2.8; (1.2–6.6)	3.1; (1.4–7.1)	2.5; (1.3–5.8)	2.8; (1.2–7.0)	2.7; (1.1–6.5)	0.39
GLIM (malnutrition) (%)	51.1	63.4	61.4	44.3	35.5	<0.001
Spot urinary creatinine concentration (g/L)	1.087	0.331	0.822	1.374	2.085	<0.001
**Comorbidities**						
Hypertension (%)	54.4	53.7	54.4	59.2	50.4	0.31
Diabetes mellitus type 2 (%)	29.4	29.1	36.8	27.2	24.6	0.03
Hypercholesterolemia (%)	59.9	59.9	60.1	61.8	57.9	0.86
Hypertriglyceridemia (%)	42.9	44.0	43.0	40.3	44.3	0.82
History of smoking (%)	73.4	72.7	71.0	70.6	79.4	0.12
**Therapy**						
ACEI/ARB (% treated)	93.1	91.2	92.5	94.7	93.9	0.003
ACEI/ARB (% of recommended dose)	50; (25–100)	50; (20–100)	50; (20–100)	50; (25–100)	50; (25–100)	0.33
BB (% treated)	97.3	97.8	96.0	97.4	97.8	0.625
BB (% target of recommended dose)	33; (25–67)	43; (25–67)	33; (25–67)	33; (25–67)	33; (33–67)	0.142
MRA (% treated)	92.2	92.5	96.0	93.0	86.8	0.003
MRA (% of recommended dose)	100; (100–200)	100; (100–200)	100; (100–200)	100; (50–100)	100; (100–100)	0.09
Loop diuretics (% treated)	87.0	90.7	86.4	88.2	82.9	0.08
Loop diuretics (mg of furosemide equivalent)	93.6 ± 75.9	105.1 ± 91.5	94.1 ± 70.8	97.4 ± 98.9	74.3 ± 61.7	<0.001
**Outcome**						
All-cause mortality at 3 years	38.7	45.0	44.5	38.7	26.7	<0.001

**Table 2:** The univariable and multivariable association of log SUCR.

Parameter	Univariable		Multivariable	
	Standard β	*p*-Value	Standard β	*p*-Value
Gender	−0.08	0.02	0.10	0.04
NYHA	−0.18	0.0001		
Systolic BP	0.11	0.001		
BMI	0.15	<0.0001		
Weight loss	−0.15	<0.0001		
Log fat tissue	0.09	0.01		
Log fat-free tissue	0.09	0.01		
Sodium	0.19	<0.0001		
NTproBNP	−0.15	<0.0001		
GLIM	−0.21	<0.0001	−0.13	0.02
Loop diuretics dose	−0.14	0.0001		
MRA percent recommended dose	0.07	0.03		

**Table 3:** The relative risk of death for any reason during follow-up–multivariable models.

	Quartiles of SUCR			
Feature	Q4 Ref.	Q3	Q2	Q1
	3-year mortality risk Cox regression analysis			
	Hazard ratio ± 95% CI, *p*-value			
Raw model	1.0	1.87; (1.23–2.85), *p* = 0.003	1.80: (1.18–2.74), *p* = 0.006	1.82; (1.20–2.78), *p* = 0.005
Model 1. adjusted for age, gender, BMI, weight loss, log fat tissue, log fat-free tissue, GLIM score, LVEF	1.0	1.58; (1.03–2.42), *p* = 0.02	1.47; (0.95–2.26), *p* = 0.08	1.66; (1.08–2.56), *p* = 0.03
Model 2 = model 1 + log loop diuretics dose + percent recommended dose of MRA	1.0	1.36; (0.87–2.12), *p* = 0.18	1.32; (0.85–2.05), *p* = 0.21	1.33; (0.85–2.08), *p* = 0.21
Model 3 = model 1 + log loop diuretics dose + percent recommended dose of MRA + serum sodium + log NTproBNP	1.0	1.34; (0.86–2.09), *p* = 0.20	1.27; (0.82–1.98). *p* = 0.29	1.20; (0.76–1.89), *p* = 0.43

## Data Availability

The data presented in this study are available on request from the corresponding author.

## Abbreviations

ICM	Ischemic etiology of HF
NYHA	New York Heart Association class
MVO2	Maximum *oxygen consumption on symptom-limited* treadmill test
LVEF	Left ventricular ejection fraction
ASMI	*Appendicular* Skeletal Muscle Index
NTproBNP	N-terminal fragment of brain-type natriuretic peptide
eGFR_MDRD_	Estimated glomerular filtration rate calculated with the use of Modification of Diet in Renal Disease formula
hsCRP	High-sensitive C-reactive protein
GLIM	The Global Leadership Initiative on *Malnutrition*
ACEI/ARB	Angiotensin-converting enzyme inhibitors/Angiotensin II receptor *blockers*
BB	Beta blockers
MRA	Mineralocorticoid receptor antagonist

## References

[1] Savarese G., Becher P.M., Lund L.H., Seferovic P., Rosano G.M.C., Coats A.J.S. (2022). Global burden of heart failure: A comprehensive and updated review of epidemiology. Cardiovasc. Res..

[2] Johansson I., Joseph P., Balasubramanian K., McMurray J.J., Lund L.H., Ezekowitz J.A., Kamath D., Alhabib K., Bayes-Genis A., Budaj A. (2021). Health-Related Quality of Life and Mortality in Heart Failure: The Global Congestive Heart Failure Study of 23 000 Patients from 40 Countries. Circulation.

[3] Moradi M., Daneshi F., Behzadmehr R., Rafiemanesh H., Bouya S., Raeisi M. (2020). Quality of life of chronic heart failure patients: A systematic review and meta-analysis. Heart Fail. Rev..

[4] Del Buono M.G., Arena R., Borlaug B.A., Carbone S., Canada J.M., Kirkman D.L., Garten R., Rodriguez-Miguelez P., Guazzi M., Lavie C.J. (2019). Exercise Intolerance in Patients with Heart Failure: JACC State-of-the-Art Review. J. Am. Coll. Cardiol..

[5] Piepoli M.F., Kaczmarek A., Francis D.P., Davies L.C., Rauchhaus M., Jankowska E.A., Anker S.D., Capucci A., Banasiak W., Ponikowski P. (2006). Reduced peripheral skeletal muscle mass and abnormal reflex physiology in chronic heart failure. Circulation.

[6] Drexler H., Riede U., Münzel T., König H., Funke E., Just H. (1992). Alterations of skeletal muscle in chronic heart failure. Circulation.

[7] Sullivan M.J., Green H.J., Cobb F.R. (1990). Skeletal muscle biochemistry and histology in ambulatory patients with long-term heart failure. Circulation.

[8] Chati Z., Zannad F., Robin-Lherbier B., Escanye J.-M., Jeandel C., Robert J., Aliot E. (1994). Contribution of specific skeletal muscle metabolic abnormalities to limitation of exercise capacity in patients with chronic heart failure: A phosphorus 31 nuclear magnetic resonance study. Am. Heart J..

[9] Cicoira M., Zanolla L., Franceschini L., Rossi A., Golia G., Zamboni M., Tosoni P., Zardini P. (2001). Skeletal muscle mass independently predicts peak oxygen consumption and ventilatory response during exercise in noncachectic patients with chronic heart failure. J. Am. Coll. Cardiol..

[10] Mancini D.M., Walter G., Reichek N., Lenkinski R., McCully K.K., Mullen J.L., Wilson J.R. (1992). Contribution of skeletal muscle atrophy to exercise intolerance and altered muscle metabolism in heart failure. Circulation.

[11] Anker S.D., Ponikowski P., Varney S., Chua T.P., Clark A.L., Webb-Peploe K.M., Harrington D., Kox W.J., Poole-Wilson P.A., Coats A.J. (1997). Wasting as independent risk factor for mortality in chronic heart failure. Lancet.

[12] Al-Absi H.R.H., Islam M.T., Refaee M.A., Chowdhury M.E.H., Alam T. (2022). Cardiovascular Disease Diagnosis from DXA Scan and Retinal Images Using Deep Learning. Sensors.

[13] Myers V.C., Fine M.S. (1913). The creatine content of muscle under normal conditions: Its relation to the urinary creatinine. J. Biol. Chem..

[14] Hargreaves M., Spriet L.L. (2020). Skeletal muscle energy metabolism during exercise. Nat. Metab..

[15] Guimarães-Ferreira L. (2014). Role of the phosphocreatine system on energetic homeostasis in skeletal and cardiac muscles. Einstein.

[16] Wallimann T., Wyss M., Brdiczka D., Nicolay K., Eppenberger H.M. (1992). Intracellular compartmentation, structure and function of creatine kinase isoenzymes in tissues with high and fluctuating energy demands: The ‘phosphocreatine circuit’ for cellular energy homeostasis. Biochem. J..

[17] Stam S.P., Eisenga M.F., Gomes-Neto A.W., van Londen M., de Meijer V.E., van Beek A.P., Gansevoort R.T., Bakker S.J. (2019). Muscle mass determined from urinary creatinine excretion rate, and muscle performance in renal transplant recipients. J. Cachex Sarcopenia Muscle.

[18] ter Maaten J.M., Maggioni A.P., Latini R., Masson S., Tognoni G., Tavazzi L., Signorini S., Voors A.A., Damman K. (2017). Clinical and prognostic value of spot urinary creatinine in chronic heart failure—An analysis from GISSI-HF. Am. Heart J..

[19] Pandhi P., Streng K.W., Anker S.D., Cleland J.G., Damman K., Dickstein K., Pellicori P., Lang C.C., Ng L., Samani N.J. (2021). The value of spot urinary creatinine as a marker of muscle wasting in patients with new-onset or worsening heart failure. J. Cachex Sarcopenia Muscle.

[20] Wilcox C.S., Testani J.M., Pitt B. (2020). Pathophysiology of Diuretic Resistance and Its Implications for the Management of Chronic Heart Failure. Hypertension.

[21] Cederholm T., Jensen G.L., Correia M.I.T.D., Gonzalez M.C., Fukushima R., Higashiguchi T., Baptista G., Barazzoni R., Blaauw R., Coats A.J. (2019). GLIM criteria for the diagnosis of malnutrition—A consensus report from the global clinical nutrition community. Clin. Nutr..

[22] Heymsfield S.B., Arteaga C., McManus C., Smith J., Moffitt S. (1983). Measurement of muscle mass in humans: Validity of the 24-hour urinary creatinine method. Am. J. Clin. Nutr..

[23] Oterdoom L.H., Gansevoort R.T., Schouten J.P., de Jong P.E., Gans R.O., Bakker S.J. (2009). Urinary creatinine excretion, an indirect measure of muscle mass, is an independent predictor of cardiovascular disease and mortality in the general population. Atherosclerosis.

[24] Ix J.H., de Boer I.H., Wassel C.L., Criqui M.H., Shlipak M.G., Whooley M.A. (2010). Urinary creatinine excretion rate and mortality in persons with coronary artery disease: The Heart and Soul Study. Circulation.

[25] Wilson F.P., Xie D., Anderson A.H., Leonard M.B., Reese P.P., Delafontaine P., Horwitz E., Kallem R., Navaneethan S., Ojo A. (2014). Urinary creatinine excretion, bioelectrical impedance analysis, and clinical outcomes in patients with CKD: The CRIC study. Clin. J. Am. Soc. Nephrol..

[26] ter Maaten J.M., Damman K., Hillege H.L., Bakker S.J., Anker S.D., Navis G., Voors A.A. (2014). Creatinine excretion rate, a marker of muscle mass, is related to clinical outcome in patients with chronic systolic heart failure. Clin. Res. Cardiol..

[27] Malinowska-Borowska J., Kulik A., Buczkowska M., Ostręga W., Stefaniak A., Piecuch M., Garbicz J., Nowak J.U., Tajstra M., Jankowska E.A. (2021). Nutritional and Non-Nutritional Predictors of Low Spot Urinary Creatinine Concentration in Patients with Heart Failure. Nutrients.

[28] Mullens W., Damman K., Harjola V.-P., Mebazaa A., Rocca H.-P.B.-L., Martens P., Testani J.M., Tang W.W., Orso F., Rossignol P. (2019). The use of diuretics in heart failure with congestion—A position statement from the Heart Failure Association of the European Society of Cardiology. Eur. J. Heart Fail..

[29] Chioncel O., Mebazaa A., Maggioni A.P., Harjola V.P., Rosano G., Laroche C., Piepoli M.F., Crespo-Leiro M.G., Lainscak M., Ponikowski P. (2019). Acute heart failure congestion and perfusion status—Impact of the clinical classification on in-hospital and long-term outcomes; insights from the ESC-EORP-HFA Heart Failure Long-Term Registry. Eur. J. Heart Fail..

[30] Valentova M., von Haehling S., Bauditz J., Doehner W., Ebner N., Bekfani T., Elsner S., Sliziuk V., Scherbakov N., Murín J. (2016). Intestinal congestion and right ventricular dysfunction: A link with appetite loss, inflammation, and cachexia in chronic heart failure. Eur. Heart J..

[31] Forman D.E., Santanasto A., Boudreau R., Harris T., Kanaya A.M., Satterfield S., Simonsick E.M., Butler J., Kizer J.R., Newman A.B. (2017). Impact of Incident Heart Failure on Body Composition Over Time in the Health, Aging, and Body Composition Study Population. Circ. Heart Fail..

[32] Post A., Tsikas D., Bakker S.J.L. (2019). Creatine is a Conditionally Essential Nutrient in Chronic Kidney Disease: A Hypothesis and Narrative Literature Review. Nutrients.

[33] Wyss M., Kaddurah-Daouk R. (2000). Creatine and Creatinine Metabolism. Physiol. Rev..

[34] Mitchell W.K., Williams J., Atherton P.J., Larvin M., Lund J.N., Narici M. (2012). Sarcopenia, Dynapenia, and the Impact of Advancing Age on Human Skeletal Muscle Size and Strength; a Quantitative Review. Front. Physiol..

[35] Sallsten G., Barregard L. (2021). Variability of Urinary Creatinine in Healthy Individuals. Int. J. Environ. Res. Public Health.

[36] Yumino D., Redolfi S., Ruttanaumpawan P., Su M.C., Smith S., Newton G.E., Mak S., Bradley T.D. (2010). Nocturnal rostral fluid shift: A unifying concept for the pathogenesis of obstructive and central sleep apnea in men with heart failure. Circulation.

[37] Redeker N.S., Adams L., Berkowitz R., Blank L., Freudenberger R., Gilbert M., Walsleben J., Zucker M.J., Rapoport D. (2012). Nocturia, Sleep and Daytime Function in Stable Heart Failure. J. Card. Fail..

[38] Carlisle T., Ward N.R., Atalla A., Cowie M.R., Simonds A.K., Morrell M.J. (2017). Investigation of the link between fluid shift and airway collapsibility as a mechanism for obstructive sleep apnea in congestive heart failure. Physiol. Rep..

[39] Okumura K., Obayashi K., Tai Y., Yamagami Y., Negoro H., Kataoka H., Kurumatani N., Saeki K. (2021). Association between NT-proBNP and nocturia among community-dwelling elderly males and females: A cross-sectional analysis of the HEIJO-KYO study. Neurourol. Urodyn..

[40] FitzGerald M.P., Litman H.J., Link C.L., McKinlay J.B. (2007). BACH Survey Investigators The Association of Nocturia with Cardiac Disease, Diabetes, Body Mass Index, Age and Diuretic Use: Results from the BACH Survey. J. Urol..

[41] Takezawa K., Kuribayashi S., Okada K., Sekii Y., Inagaki Y., Fukuhara S., Kiuchi H., Abe T., Fujita K., Uemura M. (2021). Decreased renal function increases the nighttime urine volume rate by carryover of salt excretion to the nighttime. Sci. Rep..

